# Evaluating the effect of pore size for 3d-printed bone scaffolds

**DOI:** 10.1016/j.heliyon.2024.e26005

**Published:** 2024-02-07

**Authors:** Saran Seehanam, Suppakrit Khrueaduangkham, Chomdao Sinthuvanich, Udom Sae-Ueng, Viritpon Srimaneepong, Patcharapit Promoppatum

**Affiliations:** a Center for Lightweight Materials, Design, and Manufacturing, Department of Mechanical Engineering, Faculty of Engineering, King Mongkut's University of Technology Thonburi (KMUTT), Bangmod, Bangkok, 10140, Thailand; b Department of Biochemistry, Faculty of Science, Kasetsart University, Bangkok, 10900, Thailand; c National Center for Genetic Engineering and Biotechnology (BIOTEC), National Science and Technology Development Agency (NSTDA), Pathum Thani, 12120, Thailand; d Department of Prosthodontics, Faculty of Dentistry, Chulalongkorn University, Bangkok, 10330, Thailand; e OsseoLabs Co. Ltd., Bangkok, 10400, Thailand

**Keywords:** Laser powder bed fusion process, Triply periodic minimal surface, Strut-based lattice structure, Bone scaffolds, Medical implants

## Abstract

The present study investigated the influence of pore size of strut-based Diamond and surface-based Gyroid structures for their suitability as medical implants. Samples were made additively from laser powder bed fusion process with a relative density of 0.3 and pore sizes ranging from 300 to 1300 μm. They were subsequently examined for their manufacturability and mechanical properties. In addition, non-Newtonian computational fluid dynamics and discrete phase models were conducted to assess pressure drop and cell seeding efficiency. The results showed that both Diamond and Gyroid had higher as-built densities with smaller pore sizes. However, Gyroid demonstrated better manufacturability as its relative density was closer to the as-designed one. In addition, based on mechanical testing, the elastic modulus was largely unaffected by pore size, but post-yielding behaviors differed, especially in Diamond. High mechanical sensitivity in Diamond could be explained partly by Finite Element simulations, which revealed stress localization in Diamond and more uniform stress distribution in Gyroid. Furthermore, we defined the product of the normalized specific surface, normalized pressure drop, and cell seeding efficiency as the indicator of an optimal pore size, in which this factor identified an optimal pore size of approximately 500 μm for both Diamond and Gyroid. Besides, based on such criterion, Gyroid exhibited greater applicability as bone scaffolds. In summary, this study provides comprehensive assessment of the effect of pore size and demonstrates the efficient estimation of an in-silico framework for evaluating lattice structures as medical implants, which could be applied to other lattice architectures.

## Highlights

• The present work explored the effect of pore sizes on the physical responses of strut-based Diamond and surface-based Gyroid lattice structures.
• Both lattice structures were manufactured by a laser powder bed fusion process. Gyroid showed closer relative density to the as-designed target.
• Elastic modulus was weakly dependent on pore size while post-yielding behaviors showed pore size dependency, especially in Diamond.
• Based on numerical modeling, smaller pore size increased surface area, restricted fluid transport, and boosted cell seeding efficiencies.
• An optimal pore size was determined based on the normalized specific surface, normalized pressure drop, and cell seeding efficiency.

## 1 Introduction

Medical implants have revolutionized modern healthcare, offering essential support and functionality to patients suffering from various medical conditions, including bone fractures, joint replacements, and dental prosthetics [1,2]. Over the years, the advance in design and manufacturing technologies have led to the development of innovative implant designs, including lattice structures with high porosity [3,4]. Lattice structures are characterized by their repeating unit cells with intricate geometries, offering a balance between mechanical stability and porosity [5]. The structural design of lattice implants allows for tailoring the mechanical properties and biological responses by adjusting parameters such as relative density, porous architecture, and pore size [6]. In addition, tailored mechanical properties are crucial to provide the support and stability required for bone scaffolds [7,8]. Moreover, lattice structures with interconnected porosity can facilitate nutrient diffusion and cell infiltration, promoting faster and more effective tissue regeneration [[9], [10], [11]]. Hence, understanding the influence of geometrical features on the mechanical and biological performance of these lattice structures is essential to optimize their use as medical implants.

Extensive research has been conducted on design parameters, including lattice architectures, relative density, pore size, and graded features. Such parameters have been explored for their impacts on mechanical and biological responses. For example, Wang et al. explored the biomechanical properties of Cubic, Octet, and Triply Periodic Minimal Surface (TPMS) Gyroid Ti–6Al–4V lattice structures using selective laser melting (SLM) and investigated their effects on human osteoblast-like cells [12]. The study revealed that TPMS Gyroid structures demonstrated excellent mechanical properties and biological responses, showcasing their potential as bone scaffolds. In addition, Yang et al. studied the influence of geometrical features on the mechanical properties of additively manufactured Ti–6Al–4V Gyroid [13]. Several interesting findings were reported. Firstly, under the same relative density, Gyroid exhibits excellent structural modulus when compared with other lattice counterparts, of which the results were obtained from Mazur et al. [14]. Secondly, the structural permutation of Gyroid allows the wide ranges of effective elastic modulus. They showed that Ti–6Al–4V Gyroid could be modified to match the stiffness of trabecular and cortical bones at the relative density of approximately 0.15 and 0.5, respectively. In addition to TPMS structures, Liu et al. investigated the mechanical properties of Diamond lattice structures fabricated via SLM. The study found that the Diamond lattice exhibited remarkable mechanical strength, making it viable for load-bearing orthopedic applications [15]. However, they also noted a challenge regarding manufacturing limitations and part precision. Sombatmai et al. further addressed the manufacturing limitation by performing experiments on a single strut with varied diameters from 0.3 to 2 mm [16]. The manufacturing of small struts tends to provide an oversizing result with apparent surface roughness, emphasizing the need to develop new process parameters for small parts. In addition to lattice structures with uniform features, non-uniform lattice structures have been studied by Vijayavenkataraman et al. [17] and Karuna et al. [18]. Vijayavenkataraman et al. showed the successful fabrication of graded TPMS structure. Besides, a thorough analysis from Karuna et al. showed that although lattice structure maintains a constant relative density, a graded feature can influence physical responses such as effective elastic modulus and fluid permeability. According to the previous studies, although lattice architectures can be designed and used as bone-substituting structures, the design process is highly intricate and involves several geometrical parameters.

Therefore, researchers have attempted to establish the design guideline for lattice structures, specifically for medical implant applications. Melancon et al. developed a design map for Tetrahedron-based and Octet-truss cells by considering pore size, porosity, and strut thickness [19]. The porosity mainly addresses the stiffness matching with native bones. The buildable strut thickness would reveal manufacturing limitations. The suitable pore size, on the other hand, is defined as between 50 and 650 μm for favorable bone ingrowth. In addition, Barba et al. performed a similar study on TPMS-based topology, in which several design parameters were considered for their elastic matching, manufacturability, and osseointegration [6]. In Barba et al. work, the osseointegration is dictated by the pore size between 300 and 600 μm, in which such pore size ranges are obtained from the review of published in-vivo and in-vitro studies. Although the notion of stiffness matching is agreeable for both Melancon et al. and Barba et al. desirable ranges of pore size are different. Such inconsistency could be attributed to a complex relationship between porosities, materials, pore geometries, specific surface area, and pore architectures which can affect cell proliferation and differentiation. Several studies have investigated the effect of pore size on osseointegration. Ouyang et al. designed and manufactured strut-based scaffolds with pore sizes between 400 and 1100 μm, and the porosity for all samples was kept constant at 68 % [20]. Ouyang et al. noted the competing effect among specific surface area, permeability, and shear stress, where the optimal pore size for most bone ingrowth based on in-vitro results was 650 μm. Sobral et al. utilized scaffolds from the blends of starch with poly (e-caprolactone) with both uniform and gradient pore features [21]. A significant improvement in cell seeding efficiency was noted when the gradient pore features were utilized.

Although in-vivo and in-vitro studies may reveal the effect of pore size on bone ingrowth, performing such studies requires extensive time and resources [20,22]. Thus, a comprehensive examination of vast design space is highly challenging. In this regard, numerical modeling has emerged as a design tool which could provide a rapid and resource-efficient assessment of several scaffold architectures. For example, Arjunan et al. examined six scaffold architectures using computational fluid dynamics (CFD) and Finite Element (FE) simulation [23]. The optimal scaffold was selected based on permeability, stiffness, strength, and stress concentration factors. In addition, Poltue et al. adopted the numerical simulation to establish the design guideline for TPMS-based scaffolds [24]. Effective Young's modulus, permeability, fluid-induced wall shear stress, and mechanical anisotropy were numerically simulated and considered for different TPMS architectures. Even though numerical modeling has been used to design bone scaffolds, the systematic examination of suitable pore size for medical implants is still limited.

Therefore, the present study aims to examine the effect of pore size on mechanical behavior and perform numerical assessment to indicate biological responses. Among the diverse selections of lattice structures, strut-based Diamond and surface-based Gyroid were chosen due to the significant attention they have received for their potential applications as medical implants. The objective of this study is three-fold: Firstly, to examine the manufacturability of lattice structures with varying pore sizes from laser powder bed fusion process. Secondly, to evaluate the mechanical properties of these lattice structures and analyze their mechanical sensitivity to the changes in pore size. Lastly, to interpret the biological responses of the lattice structures based on the specific surface area, pressure drop, and cell seeding efficiency using computational simulations based on non-Newtonian Computational Fluid Dynamics (CFD) and discrete phase models. In this study, the mechanical assessment would be both experimental and numerical while the investigation of fluid response would be solely numerical. The results of this study could contribute to a deeper understanding of the influence of pore size and provide valuable insights into pore size optimization.

## 2 Numerical models and experiments

### 2.1 Design of lattice structures

Strut-based and surface-based lattice structures have been proposed as potential candidates for additively manufactured bone scaffolds [12]. Specifically, the Diamond lattice was chosen to represent the strut-based structure, while the Gyroid lattice, based on the Triply Periodic Minimal Surface (TPMS) structure, was selected to represent the surface-based architecture. These choices were made due to the wide adoption of both structures for bone implant applications [15,25]. The strut-based Diamond and TPMS-based Gyroid lattices were designed using nTopology software (nTopology, New York, USA, www.ntopology.com). For brevity, we referred to the strut-based Diamond as "Diamond" and the TPMS-based Gyroid as "Gyroid" from this point onwards. The construction of the lattice structures involved assembling multiple unit cells together. To design the unit cell, various geometrical parameters need to be defined, and these parameters are known to affect the biological outcomes. The relative density, pore size, and specific surface area are often regarded as the primary factors [6,19]. In this study, the relative density was maintained at 0.3, as this value was found to provide an elastic modulus that matches well with cortical bones given Ti–6Al–4V as the base material [24]. However, the pore size varied from 300 to 1300 μm while keeping the relative density constant. Designing lattice structures with precisely defined pore sizes required setting appropriate unit cell and feature sizes. Consequently, the specific surface area was not a controlling variable but would change depending on the chosen pore size.

**Fig. 1:**
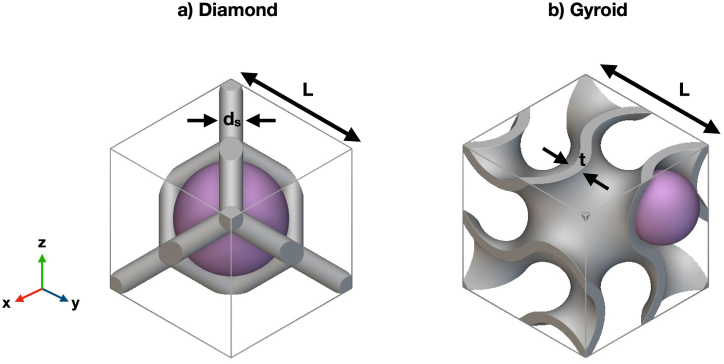
Unit cell of a) Diamond and b) Gyroid structures. Geometrical features including strut diameter (d_s_), wall thickness (t), and unit cell size (L) were shown in the figure.

The construction of Diamond and Gyroid lattice structures is discussed as follows. The unit cell of Diamond is formed by connecting 14 nodes and 16 struts, as seen in Fig. 1a [26]. The relative density of Diamond depends on the ratio of strut diameter and unit cell size, as expressed in Eq. (1) [26]. The pore size (d_pore_) is defined by the diameter of the sphere that can fit into the Diamond unit cell, as shown in Fig. 1a. In contrast to the strut-based architecture, Gyroid seen in Fig. 1b is formed by solving an implicit equation, as shown in Eq. (2). In this equation, ‘L’ represents the unit cell size, while ‘c’ is the level-set constant that can be altered to modify the offset of TPMS surfaces. The negative constant ‘c’ gives the expanding surface, whereas the positive constant ‘c’ provides the shrinking surface [27]. The solid TPMS is created by merging the shrinking and expanding surfaces, as seen in Eq. (3). Changing the ‘c’ values allows control over the solid volume of TPMS structures, where larger ‘c’ values provide the structure with a greater solid volume. By using the design guidelines from Poltue et al. [24], the relative density and the pore size of Gyroid can be determined from Eq. (4) and Eq. (5), respectively. The specific surface area (S_A_) is defined as the ratio of the surface area (A_s_) to the solid volume (V_s_), as shown in Eq. (6). The lattice structures were designed with six different pore sizes. Under these predefined pore sizes, the geometrical features of Diamond and Gyroid cells were summarized as shown in Table 1.

(1) $$\rho_{D}^{*}\propto \frac{d_{s}}{L}$$

where $\rho_{D}^{*}$ is relative density of Diamond, $d_{s}$ is strut diameter (m), and L is unit cell size (L)

(2) $$f\left(x,y,z\right)=\sin\left(\frac{2\pi }{L}x\right)\cos\left(\frac{2\pi }{L}y\right)+\sin\left(\frac{2\pi }{L}y\right)\cos\left(\frac{2\pi }{L}z\right)+\sin\left(\frac{2\pi }{L}z\right)\cos\left(\frac{2\pi }{L}x\right)=c$$

where ‘c’ is level set parameter

(3) −c≤f(x,y,z)≤c

(4) $$\rho_{G}^{*}=t\frac{3.09}{L}$$

where $\rho_{G}^{*}$ is relative density of Gyroid, and t is wall thickness (m)

(5) $$d_{\text{pore}}=0.5L-t$$

where $d_{\text{pore}}$ is pore size (m)

(6) $$S_{A}=\frac{A_{s}}{V_{s}}$$

where S_A_ is specific surface area (m^−1^), A_s_ is surface area (m^2^), and V_s_ is solid volume (m^3^).

**Table 1:** Geometrical parameters of Diamond and Gyroid lattice structures under different pore sizes.

	Diamond		Gyroid		
d_pore_ (μm)	L (mm)	d_s_ (mm)	d_pore_ (μm)	L (mm)	t (mm)
300	0.6	0.20	300	0.6	0.07
500	1.0	0.33	500	1	0.12
700	1.3	0.43	700	1.3	0.17
900	1.6	0.53	900	1.6	0.22
1100	2.0	0.67	1100	2	0.27
1300	2.4	0.80	1300	2.4	0.32

Where d_pore_ is pore size (m), L is unit cell size (m), d_s_ is a strut-thickness (m), and t is wall thickness (m).

### 2.2 Fabrication of lattice structures

Both laser-based and electron beam-based systems could be used to fabricate TPMS structures. Although the present work adopts the laser-based system, the electron beam-based work on TPMS structures was previously presented by Khrapov et al. [28]. For present study, the TRUMPF TruPrint 1000, a laser powder bed fusion (LPBF) system, was utilized to fabricate lattice samples using Ti–6Al–4V powders from AP&C GE Additive. The particle sizes ranged between 15 and 45 μm, and the chemical composition is shown in Table 2. Ti–6Al–4V was chosen for this study due to its biocompatibility and excellent corrosion resistance, making it suitable for medical applications [29]. The fabrication process occurred under low oxygen conditions with a continuous supply of argon to the build chamber. The process parameters for both Diamond and Gyroid lattices are detailed in Table 3. During post-fabrication, the samples were removed from the substrate using wire electrical discharge machining. Powder removal was accomplished through an ultrasonic bathing process, and no additional heat-treatment procedures were applied. Although the present study did not examine the effect of post-treatment processes on the mechanical responses of printed structures, they can have a noticeable impact. For example, Khrapov et al. studied the effect of surface treatments, which included a traditional powder recovery system, chemical etching, and ultrasound-based powder removal [30]. They demonstrated that different treatments could yield varying effects on residual powders, surface morphologies, grain refinement, and mechanical properties. Thus, a more thorough consideration on the impacts of post-treatment could be addressed in future studies.

**Table 2:** Chemical composition for AP&C GE Additive Ti–6Al–4V powder.

Items	Al	V	Fe	O	C	N	H	Y	Ti
**Wt. %**	6.41	4.11	0.21	0.08	0.01	0.02	0.002	<0.001	Balance

**Table 3:** LPBF process conditions of TRUMPF TruPrint 1000 for lattice samples.

Parameters	Hatching	Border
Laser power (W)	115	75
Scan velocity (mm/s)	1200	1000
Hatch spacing (μm)	80	–
Laser diameter (μm)	30	
Layer thickness (μm)	20	

**Fig. 2:**
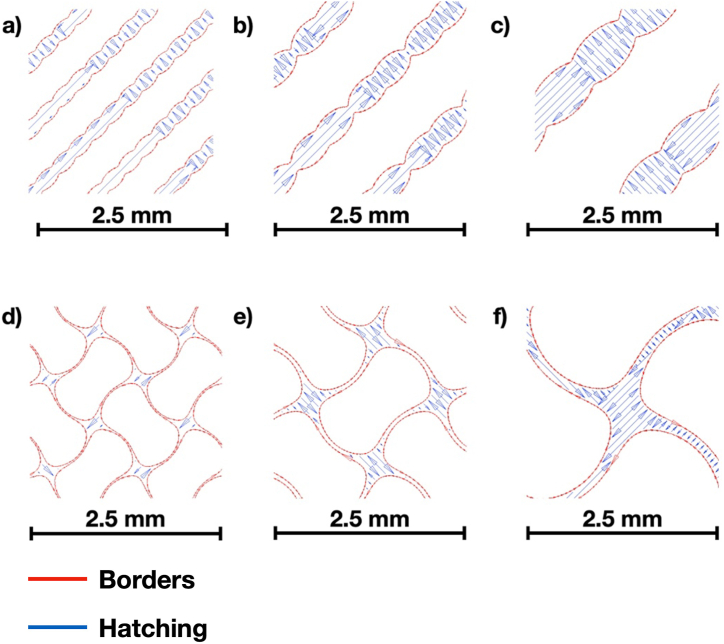
Scan patterns for a) Diamond with d_pore_ of 500 μm, b) Diamond with d_pore_ of 700 μm, c) Diamond with d_pore_ of 1300 μm, d) Gyroid with d_pore_ of 500 μm, e) Gyroid with d_pore_ of 700 μm, and f) Gyroid with d_pore_ of 1300 μm.

In addition, Fig. 2a - Fig. 2f display scan patterns for Diamond and Gyroid with the pore sizes of 500, 700, and 1300 μm. In this work, the scan trajectories can be divided into two areas: the border and hatching. According to Fig. 2a–f, the border and hatching were represented by red and blue lines, respectively. The border pattern would follow the outer contour of the 2D cross section, while the hatching pattern would fill the area inside the outer contour [31]. For hatching, chessboard patterns were adopted in the present work. Consequently, the interior hatching would be divided into islands with a size of 10 mm. Among the islands, the scan direction could be altered by 90°. As a result, one can observe in Fig. 2 that the laser pattern is alternatively parallel and perpendicular to the outer contours. Moreover, the scan pattern was kept identical between each layer. Of note, since the present work did not intend to study the effect of scan strategies, the description of the scan patterns is based on the default recommendation by the LPBF machine manufacturer. Beside, a comparison between Fig. 2a and c shows that, under larger pore sizes, the hatch region becomes greater. The same observation holds for Gyroid, as seen from Fig. 2d to f. The area of the hatch region is crucial in determining the manufacturability of the lattice structures. According to Sombatmai et al. [16], LPBF parts tend to be oversizing if the printed feature is small. Hence, manufacturing limitations should be considered while designing lattice structures with varying pore sizes.

### 2.3 Mechanical testing and simulation

A desirable medical implant should demonstrate both good mechanical integrity and biocompatibility. To explore the influence of pore sizes on mechanical responses, compression testing and FE simulation were conducted. To design specimens for mechanical testing, some experimental studies added a support layer at the top and bottom of lattice structures such as those seen in Bruson et al. [32] and Saghaian et al. [33]. Bruson et al. stated that the support layer was intended to provide uniform loading distribution [32]. However, both Bruson et al. [32] and Saghaian et al. [33], who added the support layer in their experiments, only conducted experiments on the lattice structures with a 3 × 3 × 3 cell configuration. On the other hand, many experimental studies carried out mechanical testing on the lattice structures without adding support layers, as seen in Zhou et al. [34], AlMahri et al. [35], and Köhnen et al. [36]. Additionally, Maskery et al. investigated the effect of cell configuration on the elastic responses [37]. They determined the elastic modulus of the lattice structures with cell configurations from 1 × 1 × 1 to 5 × 5 × 5. Although the elastic modulus increased with the number of unit cells, the result converged asymptotically to the upper bound values with larger cell configuration. Due to such convergence, TPMS structures without the support layer were adopted in the present study. Moreover, the experiments employed universal testing machines (Hung Ta, HT-2402) with a load cell of 100 kN, operating under quasi-static conditions. The obtained compression test results would be used to determine the elastic modulus and energy absorption, with the latter calculated using Eq. (7).

(7) $$W=\int_{0}^{\varepsilon }\sigma d\varepsilon$$

where W is energy absortion per unit volume (J/mm^3^), σ is compressive stress (MPa), and ε is compressive strain

Furthermore, while the mechanical test could provide insights into the structural responses of the lattice structure, numerical simulation can offer a deeper understanding. Therefore, FE simulation was carried out using nTopology software, considering solely the elastic response. The elastic modulus and Poisson's ratio of Ti–6Al–4V are 110 GPa and 0.3, respectively [16]. Mechanical simulations were performed to achieve two objectives. Firstly, the simulation aimed to determine the homogenized properties of the lattice structure, thereby revealing the stiffness matrix as depicted in Eq. (8). To achieve this, a numerical simulation of the unit cell was conducted using nTopology, where the homogenization module was employed to simulate various loading conditions. Consequently, the elastic anisotropy could be calculated from the stiffness matrix and was represented as the Zener ratio, $\alpha_{r}$, as shown in Eq. (9). A Zener ratio of one indicates an isotropic structure, whereas a value significantly deviating from one suggests a more anisotropic structure.

The second objective is to determine the stress distribution inside the lattice structures. Therefore, mechanical simulations were performed on structures with a 3x3x3 unit cell configuration. Two rigid planes were placed at the top and bottom of the structures. Subsequently, these structures were subjected to compressive loading in simulation, and their stress distribution was analyzed to understand the impact of lattice architectures and pore sizes. Based on the simulated mechanical responses, the outputs of interest are the effective elastic modulus, elastic anisotropy, and stress distribution for both Diamond and Gyroid structures. The elastic modulus provides valuable insights into mechanical compatibility with human bone [38]. Moreover, considering elastic anisotropy in scaffold design enables more efficient stress distribution, resulting in enhanced load-bearing capacity under complex loading conditions [39]. Additionally, the local stress distribution is a critical indicator of failure behaviors and should be duly considered [40].

(8) $$C_{\text{ij}}=\left[\begin{array}{cccccc} C_{11} & C_{12} & C_{13} & C_{14} & C_{15} & C_{16}\\ C_{21} & C_{22} & C_{23} & C_{24} & C_{25} & C_{26}\\ C_{31} & C_{32} & C_{33} & C_{34} & C_{35} & C_{36}\\ C_{41} & C_{42} & C_{43} & C_{44} & C_{45} & C_{46}\\ C_{51} & C_{52} & C_{53} & C_{54} & C_{55} & C_{56}\\ C_{61} & C_{62} & C_{63} & C_{64} & C_{65} & C_{66} \end{array}\right]$$

(9) $$\alpha_{r}=\frac{2C_{22}}{C_{11}-C_{12}}$$

### 2.4 CFD and cell seeding simulation

The fluid response and cell seeding simulation have been correlated with tissue growth within engineering scaffolds, as previously shown by Melchels et al. [41] and Liu et al. [42]. Typically, CFD analysis can reveal the fluid-structure interaction, where the physical output, such as pressure drop, can be used for the assessment of nutrient transport. The flow field from the CFD analysis is subsequently utilized for predicting cell seeding. Simulated cell seeding could be employed to determine the initial seeding efficiency for further studies on cell proliferation and differentiation.

**Fig. 3:**
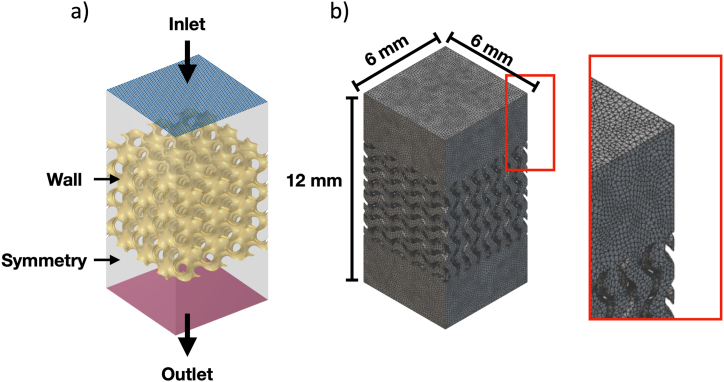
a) Numerical domain and boundary conditions for CFD simulation and b) CFD domain with meshes. The mesh type is tetrahedral element with 0.05 mm mesh size.

To obtain flow fields, the Navier-Stokes equation based on the conservation of mass and momentum, as shown in Eq. (10), was solved. Fig. 3a illustrates the computational domain, which consists of the scaffolds and flow channels. For the CFD simulation, a sample size of 6 × 6 × 6 mm³ was used, and an inlet velocity of 0.1 mm/s was chosen based on previous computational and experimental studies [43,44]. The fluid flow direction was set to be normal to the inlet surface, while the outlet was defined with a zero-gauge pressure. Solid walls were characterized by non-slip conditions, and the fluid domain walls were specified with a symmetric boundary condition. The fluid density was assumed to be 1050 kg/m³. Additionally, our previous study demonstrated that the blood viscosity model can significantly affect the fluid response [45]. This is mainly due to the complex heterogeneous mixture of human blood, which consists of red blood cells, white blood cells, and platelets combined within the liquid plasma, causing a strong shear rate dependency for blood viscosity. Therefore, instead of assuming water viscosity, we adopted the Carreau-Yasuda rheological model, as seen in Eq. (11), with the fitting parameters for the viscosity model provided in Table 4. The CFD analysis was performed using COMSOL Multiphysics software, and the computational domain with meshes can be seen in Fig. 3b. The tetrahedral mesh with an element size of 0.05 mm, resulting in a total element count of around 3,500,000, was utilized. Along with the predicted flow fields, the pressure drop was determined and compared between scaffolds with different structures and pore sizes.

(10) $$\mu \nabla^{2}\vec{u}+\rho \left(\vec{u}∙\nabla \right)\vec{u}+\nabla p=0,\nabla ∙\vec{u}=0$$

where μ is effective viscosity (Pa ∙s), $\vec{u}$ is velocity field (m/s), ρ is fluid density (kg/m^3^), ∇p is pressre drop (Pa)

(11) $$\mu =\mu_{\infty }+\left(\mu_{0}-\mu_{\infty }\right){\left(1+{\left(\lambda \dot{\gamma }\right)}^{2}\right)}^{\frac{n-1}{2}}$$

where $\mu_{0}$ is lower bound viscosity at shear rate of zero (Pa ∙ s), $\mu_{\infty }$ is upper bound viscosity at shear rate of infinity (Pa ∙ s), $\dot{\gamma }$ is shear rate (s^−1^), and λ as well as n are fitting parameters

**Table 4:** Carreau-Yasuda non-Newtonian blood viscosity models [45].

Parameters	Units	Values
$\mu_{\infty }$	Pa·s	0.0035
$\mu_{0}$	Pa·s	0.25
λ	s	69.1
n	–	0.3621

Once the predicted flow fields are obtained, the discrete phase model (DPM) is applied to reveal the interaction of cells with the flow and solid structures. The cell seeding simulation was shown to reveal the interaction between perfusion seeding and the pore architecture of the scaffold. Olivares and Lacroix studied two scaffolds with uniform and gradient pore features [9]. They found that the spatial distribution of deposited cells from the simulation was qualitatively agreeable with in-vitro results for both uniform and gradient scaffolds. Additionally, within the DPM framework, cells are represented by discrete particles, and their trajectories are influenced by fluid flows. The cell motion is governed by Newton's second law, as shown in Eq. (12) [42]. Eq. (12) is primarily affected by drag and gravitational forces, which are determined by the relative densities between the discrete and continuous phases. Furthermore, other forces, such as particle collision, are neglected. The particle relaxation time, $\tau_{r}$, can be determined using Eq. (13), which depends on fluid viscosity (μ), cell density ($\rho_{\text{cell}}$), cell diameter (d_cell_), Reynolds number (Re), and drag coefficient (C_d_). C_d_ can be calculated from Eq. (14). The Stokes number (S_tk_) was computed as shown in Eq. (15). The Stokes number can be used to determine whether the particles follow a carrier fluid streamline [46]. In Eq. (15), v denotes the average fluid velocity. d_c_ defines the characteristic dimension of the obstacles, which has a magnitude of one if the cells are assumed to be spherical [47,48]. Subsequently, the cell seeding efficiency (η_S_) can be obtained from Eq. (16), where N_i_ is the number of incoming cells, and N_t_ is the number of trapped cells within the scaffolds. The parameters for DPM used in the present work are presented in Table 5.

(12) $$\frac{du_{\text{cell}}}{\text{dt}}=\frac{\left(u_{\text{fluid}}-u_{\text{cell}}\right)}{\tau_{r}}+g\frac{\left(\rho_{\text{cell}}-\rho \right)}{\rho }$$

where $\tau_{r}$ is the particle relaxation time (s), $u_{\text{cell}}$ is cell velocity (m/s), $u_{\text{fluid}}$ is fluid velocity (m/s), g is gravitational accerelation (m/s^2^), $\rho_{\text{cell}}$ is cell density (kg/m^3^), and ρ is fluid density (kg/m^3^)

(13) $$\tau_{r}=\frac{{4\rho }_{\text{cell}}d_{\text{cell}}^{2}}{3\mu C_{d}\text{Re}}$$

where $d_{cell}$ is cell diameter, C_d_ is an empirical drag coefficient factor for spherically shaped particles, and Re is Reynolds number

(14) $$C_{d}=\frac{24}{Re}\left(1+0.15{Re}^{0.687}\right)$$

(15) $$S_{\text{tk}}=\frac{\rho_{\text{cell}}d_{\text{cell}}^{2}v}{18\mu d_{c}}$$

where S_tk_ is Stokes number, $d_{c}$ is characteristic dimension of the obstables, and v is average fluid velocity (m/s)

(16) $$\eta_{S}=\frac{N_{t}}{N_{i}}$$

where $\eta_{S}$ is cell seeding efficiency, N_t_ is the number of trapped cells, and N_i_ is the number of initial cells

**Table 5:** Parameters for discrete phase model simulation.

Parameters	Units	Values
$\rho_{\text{cell}}$	kg/m^3^	1130
$d_{\text{cell}}$	μm	10
N_i_	–	3600

### 2.5 Determination of optimum pore size

To determine the optimum pore size, we consider three aspects of additively manufactured scaffolds, including manufacturability, mechanical integrities, and biological responses. Although the present work does not include in-vitro and in-vivo test, we assess the effect of pore size on simulated cell behaviors by considering the surface area, pressure drop, and cell seeding efficiency. Typically, a high surface area and low pressure drop are preferable since they have been shown to promote better cell attachment and nutrient transport [22]. However, the interplay between these parameters has not been clearly demonstrated in the previous works. In this study, we take into account the cell seeding efficiency, as a higher cell seeding efficiency can potentially lead to better tissue formation within the scaffolds [21]. To simultaneously examine the surface area, pressure drop, and cell seeding efficiency, we normalize the specific surface area and pressure drop using their maximum and minimum values, as shown in Eqs. (17), (18), respectively. For the analysis, we combine results from both Diamond and Gyroid structures to assess the effect of pore sizes together with the influence of base architectures. Consequently, by combining Eqs. (16)–(18), we can determine the optimal pore size, as shown in Eq. (19). This will help us identify the most suitable pore size for achieving the desired biological responses in the additively manufactured scaffolds.

(17) $$\eta_{A}=\frac{S_{A}-S_{A,\min}}{S_{A,\max}-S_{A,\min}}$$

where $\eta_{A}$ is normalized specific surface area

(18) $$\eta_{P}=\frac{{\Delta P}_{\max}-\Delta P}{{\Delta P}_{\max}-{\Delta P}_{\min}}$$

where $\eta_{P}$ is normalized pressure drop

(19) $$\eta =\eta_{A}\times \eta_{P}\times \eta_{S}$$

where η is a combination of normalized specific surface area, normalized pressre drop, and cell seeding efficiency to identify optimal pore size

## 3 Results and discussions

### 3.1 Surface characteristics and manufacturability of lattice structures

**Fig. 4:**
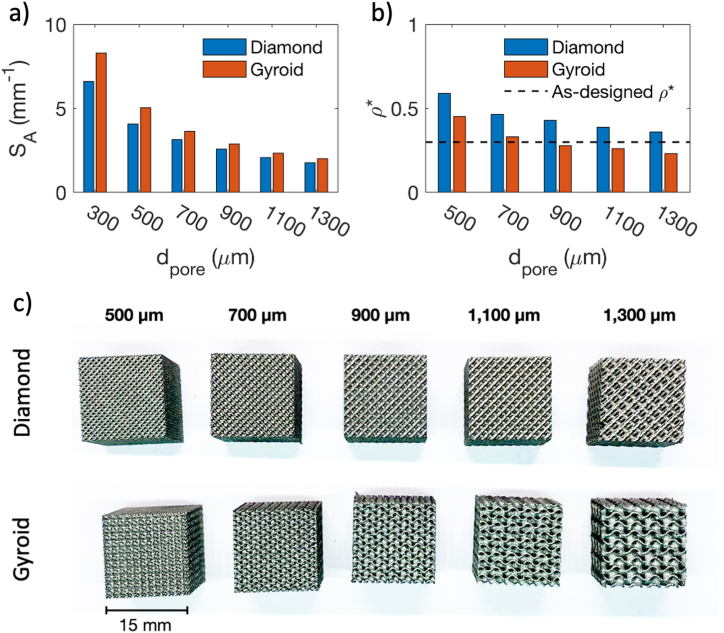
a) Specific surface area of lattice structure with different pore sizes, b) Comparison between as-designed and as-built relative densities of Diamond and Gyroid, and c) Additively manufactured Diamond and Gyroid with pore sizes from 500 to 1300 μm, and.

Fig. 4a showed the dependency of specific surface area (S_A_) on pore size (d_pore_) of Diamond and Gyroid structures. As depicted in Fig. 4a, the S_A_ can increase by around three to four times when the pore size (d_pore_) changes from 1300 to 300 μm. Both Diamond and Gyroid exhibit a similar trend, with S_A_ increasing as d_pore_ becomes smaller. However, Gyroid generally has a higher S_A_ than Diamond. Specifically, when considering d_pore_ of 300 and 1300 μm, Gyroid's S_A_ was higher than Diamond's by 25% and 13%, respectively. The interconnected surface of the Gyroid structures provides a higher surface area than their strut-based counterpart. On the other hand, Fig. 4b compares the as-designed and as-built relative densities. Despite both Diamond and Gyroid being designed with the same relative density of 0.3, Diamond resulted in generally greater relative densities than Gyroid. Additionally, printed structures tended to be denser with smaller pore sizes. Changing d_pore_ from 500 to 1300 μm, the relative density changes from 0.59 to 0.36 for Diamond and from 0.45 to 0.23 for Gyroid, respectively. Even though the resulting densities varied with d_pore_ for both structures, Gyroid exhibited better manufacturability than Diamond since the resulting relative density is closer to the targeted value. It is important to note that the manufacturability of LPBF process relies on both the structures and process parameters. Although only default parameters were explored in the present work, Fig. 4b emphasize the need to develop a new set of process guidelines, specifically aimed at samples with small geometrical features. An example of such work can be seen in Xue et al. who explored single contour scan strategies to improve the printing accuracy of Ti–6Al–4V struts [49]. Nonetheless, Fig. 4c also showed that Diamond and Gyroid with d_pore_ ranging from 500 to 1300 μm could be printed successfully without apparent cracks on the structures.

### 3.2 Compression test results

**Fig. 5:**
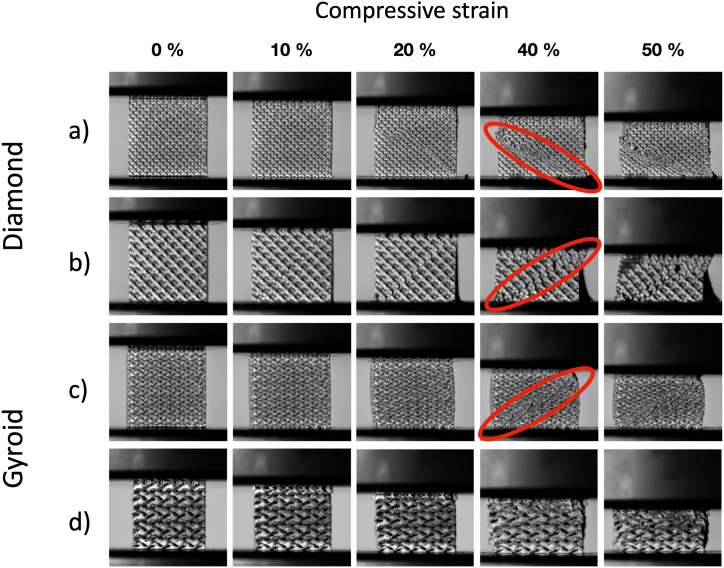
In-situ images during compression testing of a) Diamond cell sample with d_pore_ of 700 μm, b) Diamond cell sample with d_pore_ of 1300 μm, c) Gyroid cell sample with d_pore_ of 700 μm, d) Gyroid cell sample with d_pore_ of 1300 μm. A red circle highlights an example of macroscopic shear band. (For interpretation of the references to colour in this figure legend, the reader is referred to the Web version of this article.)

Lattice samples were subjected to compressive test and FE analysis to determine the effect of pore sizes on mechanical responses. The compression test was performed to determine the effect of pore size on elastic modulus, initial peak stress, and energy absorption. Besides, to provide more information on mechanical responses, the numerical simulation considered additional aspects of local stress distribution and elastic anisotropy. Based on the observation from Fig. 4b, where samples with d_pore_ of 500 μm showed resulting density significantly deviating from the as-designed value, only samples with d_pore_ ranging from 700 to 1300 μm were examined in the experiments. Fig. 5a - Fig. 5d illustrate the deformation of lattice structures during compression tests. From Fig. 5a and Fig. 5b, it can be observed that the macroscopic shear band for Diamond was visible at a compressive strain of 20 %. As the strain increased, the shear band became more pronounced. Notably, Diamond with a larger pore size displayed a more catastrophic collapse compared to one with a smaller pore size. Furthermore, Fig. 5c shows that Gyroid with d_pore_ of 700 μm can also exhibit a macroscopic shear band, but it occurred at a much larger strain compared to Diamond. However, with d_pore_ of 1300 μm, the shear band in Gyroid became much less apparent, indicating improved mechanical stability, as seen in Fig. 5d.

**Fig. 6:**
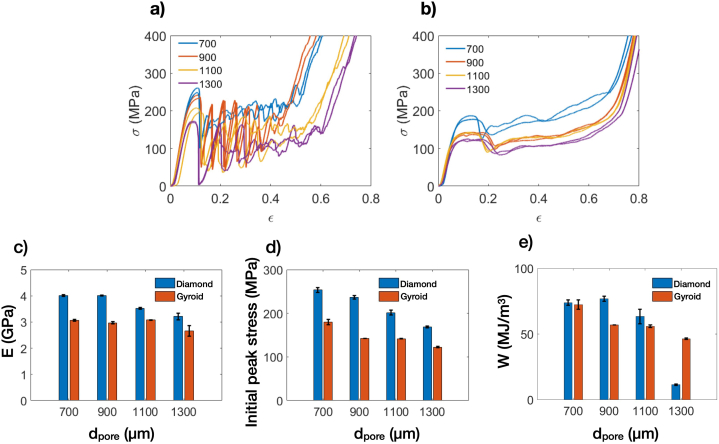
a) Stress-strain responses for Diamond with d_pore_ between 700 and 1300 μm, b) Stress-strain responses for Gyroid with d_pore_ between 700 and 1300 μm, c) Elastic modulus of Diamond and Gyroid, d) Initial peak stress of Diamond and Gyroid, and e) Volumetric energy absorption of Diamond and Gyroid.

The stress-strain responses from compression test for both Diamond and Gyroid structures are presented in Fig. 6a and b, respectively. Two identical tests were performed for each sample configuration, and good repeatability was demonstrated. In Fig. 6a, the stress-strain response of Diamond exhibited strong fluctuations, which became more apparent at larger d_pore_ values. Conversely, for Gyroid, as seen in Fig. 6b, the fluctuation in stress responses seemed relatively subtle, regardless of d_pore_. From the compression test results, the elastic modulus, initial peak stress, and energy absorption were determined for each sample, as shown in Fig. 6c to Fig. 6e, respectively. The elastic modulus and initial peak stress were determined based on the slope of the linear response and the maximum stress at the first stress collapse. And the energy absorption was calculated based on Eq. (7). Fig. 6c displays the elastic modulus for both Diamond and Gyroid, with Diamond exhibiting a higher modulus than Gyroid. Moreover, the elastic modulus for Gyroid did not change noticeably under different d_pore_ values. However, for Diamond, the elastic modulus increased by 10% when d_pore_ was reduced from 1300 to 700 μm. This change in elastic modulus could be influenced by the variation in as-built relative density, as shown previously in Fig. 4c. Largely, the experimental results indicate that the elastic modulus is mainly influenced by the sample architecture with minimal effect of d_pore_. Besides, as seen in Fig. 6d, the initial peak stress increases with decreasing d_pore_. Nonetheless, Diamond exhibits much greater sensitivity to d_pore_ compared to Gyroid. For example, between d_pore_ values of 700 and 1300 μm, the initial peak stress reduces from 254 to 169 MPa for Diamond and 180 to 123 MPa for Gyroid, representing an approximate change in stress of 50 and 30 %, respectively. Additionally, Fig. 6e showed that the energy absorption is also highly sensitive to d_pore_. The energy absorption varied between 11 and 75 MJ/m³ and 47–73 MJ/m³ for Diamond and Gyroid, respectively. Overall, the compressive results reveal the significant sensitivity to d_pore_, especially for post-yielding behavior of Diamond.

### 3.3 Mechanical simulation results

**Fig. 7:**
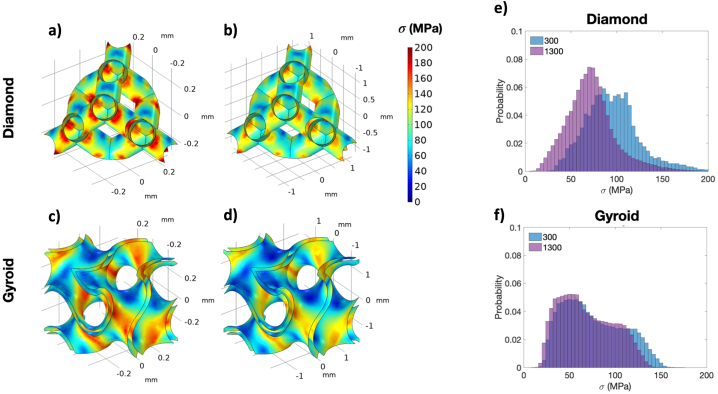
Stress distribution of a) Diamond cell with d_pore_ of 300 μm, b) Diamond cell with d_pore_ of 1300 μm, c) Gyroid cell with d_pore_ of 300 μm, d) Gyroid cell with d_pore_ of 1300 μm, and stress distribution of e) Diamond cell with d_pore_ of 300 and 1300 μm, and f) Gyroid cell with d_pore_ of 300 and 1300 μm.

**Fig. 8:**
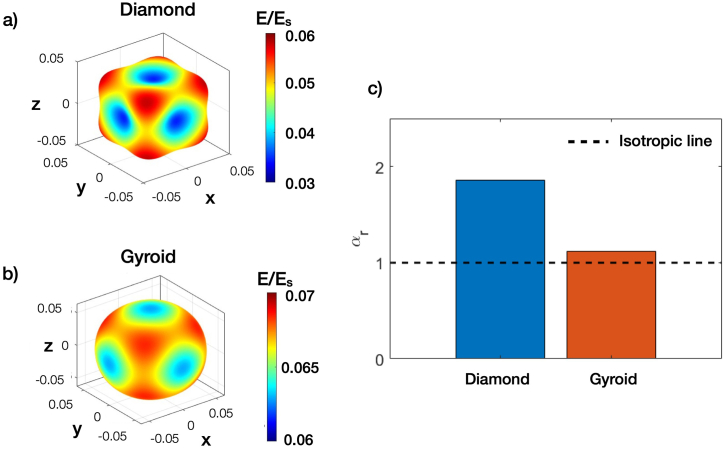
Young's modulus surface of a) Diamond and b) Gyroid, and c) Zener ratio, $\alpha_{r}$, of Diamond and Gyroid.

Following the compression test, Finite Element (FE) analysis of lattice structures with a 3 × 3 × 3 cell configuration was performed. Simulated results were evaluated for the cells in the middle of the structures. Stress fields in such cells were shown in Fig. 7a to Fig. 7d for Diamond with d_pore_ of 300 μm, Diamond with d_pore_ of 1300 μm, Gyroid with d_pore_ of 300 μm, and Gyroid with d_pore_ of 1300 μm, respectively. In addition, local stresses at all mesh nodes of the middle cells were plotted for their distribution as shown in Fig. 7e and Fig. 7f for Diamond and Gyroid structures, respectively. In Fig. 7a and Fig. 7b, high-stress concentration was observed at nodes between struts for Diamond structures. However, the magnitude of the stress at nodes became slightly higher with a smaller d_pore_, as seen in Fig. 7e. On the contrary, when considering the stress contours of Gyroid in Fig. 7c and Fig. 7d, the stress appears to be distributed more uniformly. Additionally, Fig. 7f reveals that the stress distribution in the Gyroid does not change significantly with d_pore_. Overall, the analysis from Fig. 7a to Fig. 7f provides valuable insights into the high sensitivity of post-yielding behaviors of Diamond structures, which may be explained by highly localized stress at strut joints. Additionally, based on numerically homogenized results of the unit cell, Fig. 8a and Fig. 8b display Young's modulus surface of Diamond and Gyroid, respectively. By examining the modulus surface, it becomes evident that the Gyroid exhibits more isotropic characteristics than the Diamond. From Fig. 8c, the Zener ratio of Diamond is closer to two, while that of Gyroid is slightly higher than one, indicating more isotropic behavior of Gyroid than Diamond.

### 3.4 Assessment of fluid response and cell seeding

**Fig. 9:**
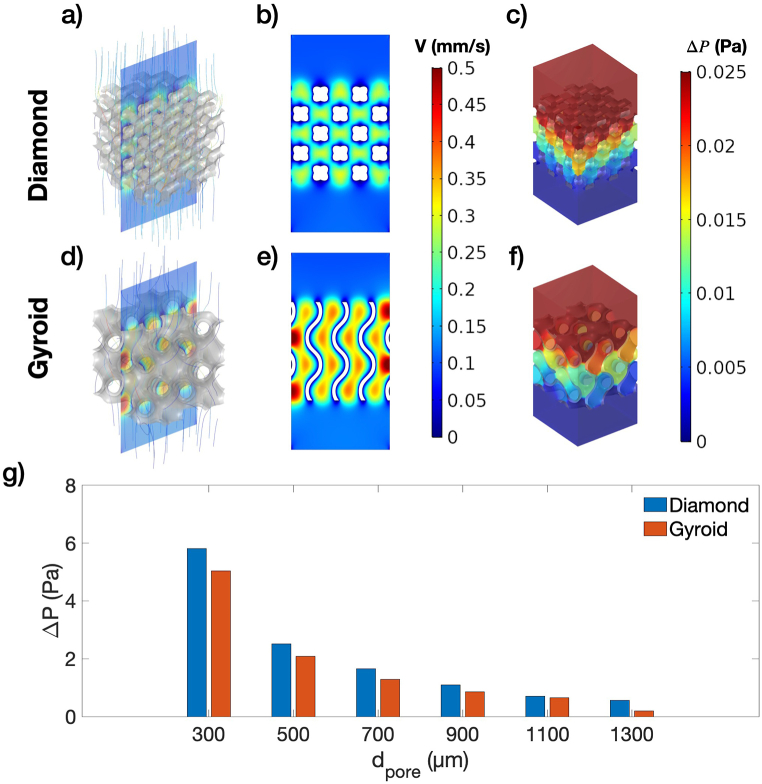
a) Fluid streamline of Diamond, b) Velocity contour of Diamond, c) Pressure contour of Diamond, d) Fluid streamline of Gyroid, e) Velocity contour of Gyroid, f) Pressure contour of Gyroid, and g) Pressure drop for Diamond and Gyroid with d_pore_ between 300 and 1300 μm. Samples shown in the figure are with d_pore_ of 1300 μm.

CFD analysis was performed to study the fluid flow behavior within the scaffolds. Fig. 9a - Fig. 9f illustrate the velocity streamlines, velocity contours, and pressure contours for Diamond and Gyroid with a d_pore_ of 1300 μm. Both geometries exhibited complex streamlines, and the lattice architecture profoundly influenced velocity profiles, as seen in Fig. 9b and Fig. 9e. Additionally, pressure drop contours were shown in Fig. 9c and Fig. 9f, and a comparison of pressure drops for both lattice structures is presented in Fig. 9g. As shown in Fig. 9g, despite both samples having a constant relative density, the pressure drop increases exponentially with smaller d_pore_. Decreasing d_pore_ from 1300 to 300 μm, the pressure drop increases from 0.57 to 5.81 Pa for Diamond and 0.52–5.04 Pa for Gyroid. Such results indicate that lattice structures with small d_pore_ can result in poor nutrient transport and cell blockage due to excessive flow resistance. Moreover, under the same d_pore_, Gyroid shows a smaller pressure drop than Diamond, approximately 10–20 %. Overall, these findings emphasize the importance of d_pore_ when designing lattice structures for optimal nutrient transport.

**Fig. 10:**
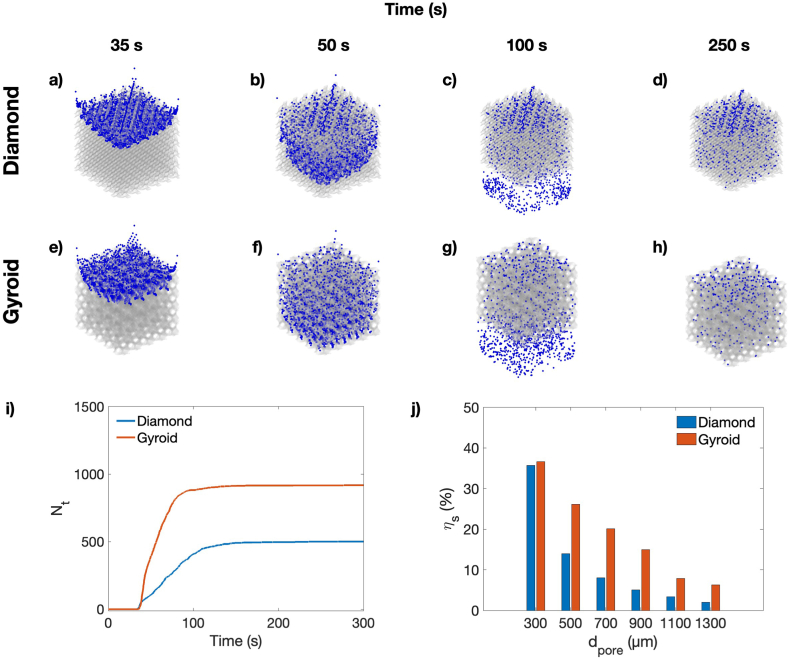
Cell seeding simulation at different time steps for (a–d) Diamond and (e–h) Gyroid, i) Number of trapped cells over time for Diamond and Gyroid with d_pore_ of 500 μm, and j) Cell seeding efficiency for Diamond and Gyroid under d_pore_ between 300 and 1300 μm.

Besides, based on simulated streamlines, the discrete phase model (DPM) was subsequently used to determine the cell seeding efficiency. As cells pass through the scaffolds, they can deposit or continue passing through. Fig. 10a, Fig. 10b, Fig. 10c, and Fig. 10d show the cell positions for Diamond at different time points of 35, 50, 100, and 250 s, respectively. On the other hand, Fig. 10e, Fig. 10f, Fig. 10g, and Fig. 10h also show the cell positions at the same time points for the Gyroid scaffolds. After 250 s, the cell deposition reaches saturation. Fig. 10d and Fig. 10h show that cells are distributed throughout the lattice scaffolds. However, a high-density deposition is observed at a frontal region. Furthermore, the numbers of trapped cells on the lattice surfaces are presented in Fig. 10i for Diamond and Gyroid with a d_pore_ of 500 μm. Fig. 10i indicates that Gyroid reaches the saturated stage more quickly than Diamond. Additionally, Fig. 10j reveals that as the pore size decreases, the number of adhered cells increases, resulting in a greater cell seeding efficiency. Within the d_pore_ range of 300–1300 μm, the seeding efficiency varied approximately between 2 and 35 % for Diamond and 6 and 36 % for Gyroid, respectively. By comparing both porous architectures, Gyroid exhibited a higher number of adhered cells. This is hypothesized to be due to Gyroid's larger specific surface area, which increases the chances of cell attachment to the scaffold's surfaces. The DPM analysis highlights the differences in cell seeding efficiency between both scaffolds and suggests that the unique porous architecture of Gyroid contributes to superior cell seeding efficiency.

### 3.5 Comprehensive evaluation of pore size

**Fig. 11:**
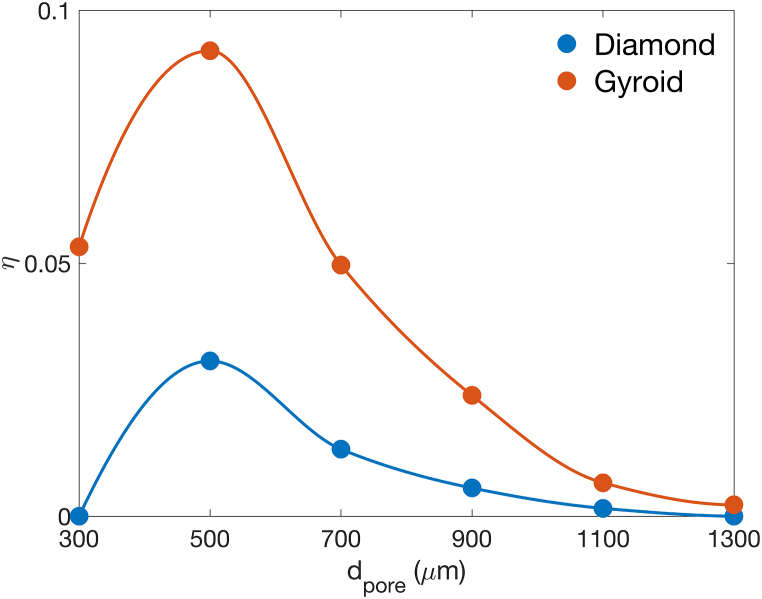
Simultaneous assessment on the effect of pore sizes and lattice architectures on specific surface area, pressure drop, and cell seeding efficiency.

The influence of d_pore_ on surface area, flow characteristics, cell seeding efficiency, and mechanical responses has been explored. Thus far, it is evident that changing d_pore_ can result in competing effects with various parameters. For example, while the surface area and cell seeding efficiency increase with a smaller d_pore_, the pressure drop becomes much higher. Additionally, both strut-based and surface-based structures exhibit a similar dependency on d_pore_, but the magnitude of physical outputs may differ. As a result, the specific surface area and pressure drop were normalized, as defined in Eqs. (17), (18). Subsequently, η, which is the product of the normalized specific surface, normalized pressure drop, and cell seeding efficiency, was determined and shown in Fig. 11. According to Fig. 11, the combined parameter indicates the optimal d_pore_ to be approximately 500 μm for both Diamond and Gyroid. Interestingly, this calculated optimal pore size aligns well with previously published works. For example, Barba et al. proposed the design map for TPMS-based synthetic bone structures [6] and identified the optimal pore size for the osseointegration aspect to be around 300–600 μm. Barba et al. mentioned the competing effect between osteoblast cell colonization and vascularization from different pore sizes. Generally, a larger d_pore_ could be more favorable for vascularization [50]. Additionally, Fukuda et al. examined the osteoinduction of porous titanium implants [51]. They fabricated additively manufactured porous implants with d_pore_ varying between 500 and 1200 μm and found excellent osteoinduction at d_pore_ of 500 μm. A similar result was also shown by Taniguchi et al. who performed in-vivo testing of porous implants [52]. They designed the scaffolds with a constant relative density and d_pore_ varying from 300, 600, and 900 μm. They found that the scaffold with d_pore_ of 600 μm exhibited greater fixation strength than those with d_pore_ of 300 and 900 μm, displaying better osseointegration of 600 μm pore size. Moreover, the comparison between Diamond and Gyroid from Fig. 11 suggests that the surface-based architecture is more suitable for bone scaffolds than the strut-based one. This is because η of Gyroid was greater than that of Diamond under the same d_pore_. Such comparison aligns well with experimental work from Wang et al. [12], who reported a superior biomechanical property of Gyroid compared to Cubic and Octet struts. In in-vitro testing, Gyroid exhibited higher cell viability than both strut architectures. By comparing our findings with previous in-vitro and in-vivo studies, the modelling framework proposed herein could provide a reasonable assessment of the suitability of lattice scaffolds for use as medical implants. With in-silico based methods, future studies on scaffolds with different architectures could be conducted with significantly reduced time and resource consumption. However, while Fig. 11 identifies a suitable d_pore_ of 500 μm, manufacturability could be a concern. As shown in Fig. 4b, additively manufactured lattice structures with small d_pore_ can deviate greatly from the as-designed model. Therefore, the development of process parameters targeted specifically at structures with small d_pore_ should be addressed more thoroughly in a future study.

## 4 Outlook for future studies

A previous study showed that Ti–6Al–4V Gyroid could be modified so that the stiffness could closely match that of trabecular and cortical bones [13]. The present work further demonstrates that other geometric features, such as pore size, can be adjusted to achieve different fluid and cell seeding responses while maintaining the same relative density. Although these findings are fundamentally essential for developing porous-based medical devices, it is critical to assess the limits of implant size and porous features in order to successfully incorporate lattice structures into medical devices. The implant size could vary depending on the types of devices, the size of bony defect, and anatomy. For example, a porous-based dental implant can be as small as 0.4 cm [53], whereas porous-based orthopedic devices, such as sacral reconstructions, could be as large as 30 cm [54]. Therefore, in order to address the limits of implant size and porous architectures, a designated application may need to be identified, in which other critical aspects such as load-bearing conditions [55] and surgical procedure [56] can be taken into consideration.

In addition, although the present work utilizes in-silico modeling to provide an initial screening of different porous structures, it does not attempt to provide biological assessments without performing biological testing. While the present work considered variables such as relative density, pore size, mechanical responses, and fluid behaviors, many other parameters could affect biological outcomes, and they were not addressed in this study. For instance, Kim et al. examined the biological properties of 3D-printed titanium [57]. They investigated three surface morphologies, including as-printed, sandblasted, and machined surfaces. Despite using identical materials, Kim et al. demonstrated that different roughness levels affected wettability and cell morphologies. Therefore, even though there are existing in-vitro and in-vivo studies on the effect of pore size on bone ingrowth [58], future research may concentrate on the synergistic or competing effects between pore size and surface characteristics, wherein various surface treatments may be applied to additively manufactured porous samples prior to biological assessment.

## 5 Conclusion

The present work examined the effect of pore size on strut-based Diamond and surface-based Gyroid on their suitability as medical implants. Samples were designed with a constant relative density of 0.3 and pore sizes varying between 300 and 1300 μm. Three primary aspects were assessed, including manufacturability, mechanical properties, and biological responses. Besides, the present work interprets the biological responses based on physical outputs, which include specific surface area, pressure drop, and cell seeding efficiency. A numerical simulation of pressure drop and cell seeding efficiency was based on a non-Newtonian CFD model and a one-way coupling discrete phase model, respectively. The conclusion could be found as follows:

1. Under the process parameters of the laser powder bed fusion machine adopted in the present work, both Diamond and Gyroid showed greater as-built density with smaller pore size. However, Gyroid exhibits better manufacturability than Diamond due to the resulting relative density, which is closer to as-deigned density.
2. From compression testing, the elastic modulus does not change significantly with pore size. However, the post-yielding behaviors, such as initial peak stress and energy absorption, show a noticeable dependency on pore size. However, mechanical sensitivity with pore size is more discernible in Diamond.
3. From FE simulation, stress localization was observed at nodes of a strut-based structure. A more uniformly distributed stress was seen for surface-based Gyroid. Different stress distribution could explain distinct difference of post-yielding behaviors between both structures.
4. Changing pore size can result in competing effects among different physical outputs. Decreasing pore size could increase the specific surface area, restrict fluid transport, and indicate the potential increase of cell seeding efficiency.
5. We defined the product of the normalized specific surface, normalized pressure drop, and cell seeding efficiency as the indicator of an optimal pore size. We found that the optimal pore size was approximately 500 μm for both Diamond and Gyroid. Nonetheless, based on a specified criterion, Gyroid exhibits more superiority as biological scaffolds.
6. Overall, the present work shows that an in-silico-based framework could provide prediction, which agrees well with in-vitro and in-vivo studies. As a result, such a framework could be extended to provide a quick and efficient estimate of other lattice architectures for their suitability as medical implants.

## Data availability statement

Data will be made available on request.

## Additional information

No additional information is available for this paper.

## CRediT authorship contribution statement

**Saran Seehanam:** Writing – review & editing, Writing – original draft, Visualization, Validation, Software, Resources, Methodology, Investigation, Formal analysis, Data curation. **Suppakrit Khrueaduangkham:** Writing – review & editing, Writing – original draft, Visualization, Validation, Resources, Methodology, Investigation, Formal analysis, Data curation. **Chomdao Sinthuvanich:** Writing – review & editing, Writing – original draft, Investigation. **Udom Sae-Ueng:** Writing – review & editing, Writing – original draft, Funding acquisition. **Viritpon Srimaneepong:** Writing – review & editing, Writing – original draft, Investigation, Funding acquisition, Conceptualization. **Patcharapit Promoppatum:** Writing – review & editing, Writing – original draft, Supervision, Project administration, Methodology, Investigation, Funding acquisition, Formal analysis, Data curation, Conceptualization.

## Declaration of generative AI and AI-assisted technologies in the writing process

During the preparation of this work, the authors used ChatGPT in order to improve readability. After using this tool, the authors reviewed and edited the content as needed and take full responsibility for the content of the publication.

## Declaration of competing interest

The authors declare that they have no known competing financial interests or personal relationships that could have appeared to influence the work reported in this paper.
